# Epigastralgies révélant un lymphome pancréatique primitif à grandes cellules B chez un patient jeune: à propos d’un cas

**DOI:** 10.11604/pamj.2018.31.161.16850

**Published:** 2018-11-02

**Authors:** Benmoussa Amine, Lasri Najat, Tazi Ilias

**Affiliations:** 1 Service d’Hématologie CHU Mohammed VI Marrakech, Faculté de Médecine et de Pharmacie Cadi Ayyad, Maroc

**Keywords:** Lymphome pancréatique, lymphome non hodgkinien à grandes cellules B, pancréatite, Pancreatic lymphoma, large B-cell non-Hodgkin’s lymphoma, pancreatitis

## Abstract

Le lymphome malin non hodgkinien (LMNH) primitif du pancréas est une localisation extrêmement rare des LMNH extraganglionnaires représentant moins de 0,7% de tous les LMNH et moins de 0,5% des tumeurs malignes pancréatiques, touchant essentiellement le sujet âgé et très rarement le sujet jeune (l'intérêt de notre cas). Son diagnostic est difficile simulant cliniquement l'adénocarcinome de pancréas et les pancréatites chroniques. Nous rapportons le cas d'un patient de 25ans, suivi pour lymphome non hodgkinien (LNH) diffus à grandes cellules B de localisation pancréatique primitive révélé par des épigastralgies avec ictère foudroyant d'installation brutale dans un contexte d'altération de l'état général et diagnostiqué sur une biopsie de la loge duodéno-pancréatique. Le diagnostic et la prise en charge précoce de ces tumeurs agressives permettent d'améliorer significativement leur pronostic.

## Abstract (en)

Primitive malignant pancreatic non-Hodgkin’s lymphoma (NHL) is an extremely rare site of extranodal NHL, accounting for less than 0.7% of all NHLs and less than 0.5% of malignant pancreatic tumors. It mainly affects the elderly and very rarely the young subject (hence the interest in our case). Clinical diagnosis is difficult because it mimics pancreatic adenocarcinoma and chronic pancreatitis. We report the case of a 25 year old patient, treated for diffuse primary pancreatic large B-cell NHL revealed by epigastralgia with fulminating jaundice characterised by acute onset associated with alteration of the general state. NHL was diagnosed by biopsy of the duodeno-pancreatic cavity. Diagnosis and early treatment of these aggressive tumors enable remarkable improvements in prognosis.

## Introduction

Les lymphomes non hodgkiniens (LNH) constituent un groupe hétérogène des tumeurs malignes de tissu lymphoïde et représentent 4% de tous les cancers [1]. Les LNH extra ganglionnaires représentent 30% des LNH, les localisations les plus fréquentes sont digestives, cutanées et pulmonaires. La localisation pancréatique primitive est rare et son diagnostic est difficile du fait de l'absence de signes cliniques et radiologiques spécifiques, reposant sur les données anatomopathologiques d'une biopsie pancréatique. Le traitement repose sur la chimiothérapie et le pronostic dépend de la précocité de la prise en charge.

## Patient et observation

Il s'agissait d'un patient âgé de 25 ans, qui s'est présenté pour une symptomatologie d'installation rapidement progressive évoluant depuis un mois, faite des épigastralgies, avec ictère intense dans un contexte d'altération de l'état général (amaigrissement chiffré a 3kg/mois; asthénie; anorexie; sans notion de fièvre prolongée ni de sueurs nocturnes). L'examen clinique trouvait un patient avec PS (performance staturale) de 2, stable sur le plan neurologique, hémodynamique et respiratoire, ictère généralisé, le reste de l'examen somatique était sans particularités, notamment pas d'adénopathies ni hépatosplénomégalie. La Tomodensitométrie (TDM) abdominale a montré la présence d'un processus tumoral de la tête de pancréas hypodense prenant le produit de contraste et mesurant 82 mm (antéropostérieur) x 57mm (épaisseur) avec dilatation de canal de Wirsung qui mesure 6,5mm et des voies biliaires principales de 16mm, extension locorégionale avec envahissement vasculaire de la veine mésentérique supérieure et l'artère hépatique. L'écho-endoscopie bilio-pancréatique montrait une volumineuse lésion hypoéchogène hétérogène de la tête de pancréas responsable d'un rétrécissement duodénal empêchant la progression de l'écho-endoscope, l'exploration était incomplète. Le patient a bénéficié d'une biopsie de la loge pancréatico-duodénale qui était en faveur de LNH diffus à grandes cellules B CD20+; KI67 à 90% (2 lectures dans deux laboratoires différents pour éliminer un éventuel lymphome de burkitt).

**Figure 1:**
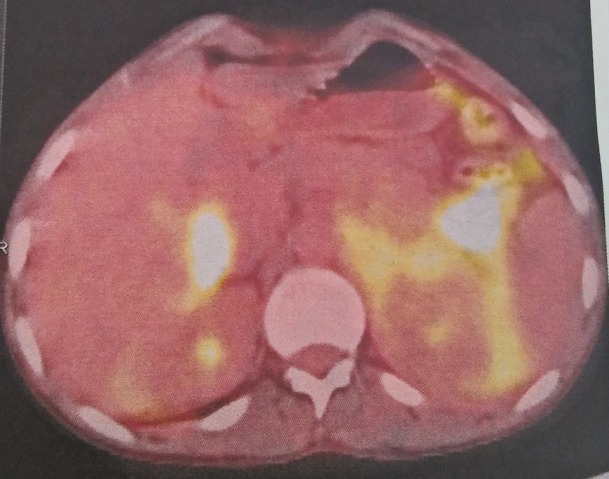
TEP scan de fin de traitement objectivant des foyers hyper métaboliques au niveau pancréatique et hépatique

Le bilan d'extension (la TDM cervico-thoraco-abdomino-pelvienne, la biopsie ostéomédullaire, hémogramme, urée, créatinine, aspartate aminotransférase, alanine transaminase) était normal ainsi que le bilan préchimiothérapie (écho-cœur et les sérologies VIH, VHC, VHB). La LDH était augmentée à 370 UI, la lipasémie élevée à 110 UI l et l'amylasémie était à 150 UI L. Le patient a été mis sous protocole RCHOP (Rituximab, Endoxan, Cyclophosphamide, Prednisolone, Doxorubicine). Le bilan d'évaluation à mi-chemin après 4 cures a montré une régression de 90% de la masse tumorale pancréatique. La Tomographie par Émission de Positrons (TEP) scanner demandée en fin de traitement (après 8 cures) objectivait des foyers hypermétaboliques sous diaphragmatiques en faveur de maladie évolutive (score de Deauville 5) (Figure 1). Le malade est actuellement sous traitement de deuxième ligne RDHAP (Rituximab, Dexamethasone, Aracytine HD, Carboplatine).

## Discussion

**Le LNH primitif du pancréas** se définit par une atteinte limitée au pancréas et aux adénopathies péri-pancréatiques sans notion d'adénopathies superficielles ou profondes, ni hépatosplénomégalie, avec l'absence de leucémisation au moment de diagnostic [2]. La localisation primitive de lymphome pancréatique est beaucoup moins rare que l'atteinte secondaire à une forme systémique. Il survient avec prédilection chez le sujet âgé de plus de 60 ans ou chez les malades avec des syndromes d'immunodéficience (VIH: 5% des cas, ou greffe d'organe) avec une prédominance masculine (sexe ratio: 1,9) [3]. Ce n'est pas le cas dans notre observation. **La symptomatologie clinique** est non spécifique faite essentiellement de [4,5]: douleur abdominale (83%), masse abdominale (58%), perte de poids très manifeste (50%), ictère (42%) car la tête du pancréas est le site le plus atteint: plus de 80%. D'autres signes peuvent être observés [6]: nausées, vomissements, diarrhée, fièvre. Les frissons et les sueurs nocturnes sont rarement observés dans les lymphomes pancréatiques primaires. Notre patient avait des épigastralgies, ictère avec altération de l'état général. **Aucun signe biologique** n'est spécifique, le lactate déshydrogénase et B2-microglobuline peuvent être élevés. Le taux de CA19-9 chez les patients atteints de LNH pancréatique est normal ou légèrement élevé, un taux élevé de CA19-9 est fortement évocateur d'un adénocarcinome pancréatique (80% des patients) [7, 8].

**Les examens radiologiques** permettent d'orienter vers le diagnostic de LNH primitif pancréatique (LPP) [8]: la tomodensitométrie abdominale peut montrer deux images caractéristiques [9]: une masse tumorale localisée et bien circonscrite avec un rehaussement homogène retardé sans infiltration vasculaire rarement avec dilatation de canal pancréatique; ou un élargissement diffus infiltrant ou remplaçant une grande partie du pancréas; Le PET-scan joue également un rôle important dans la démarche diagnostique et le suivi des LPP [10,11] peut montrer des foyers d'hyperfixation nodulaires, segmentaires ou diffus, témoin d'hypermétabolisme. Les foyers d'hypermétabolisme sont plus manifestes dans les LPP que dans l'adénocarcinome (ADK) pancréatique [12]; IRM pancréatique objective souvent une masse hypo intense en T1 localisée ou diffuse, d'intensité variable en T2, prenant le produit de contraste de façon homogène [13]; Cholangio-pancréatographie rétrograde endoscopique peut mettre en évidence [14]: (sténose légère du canal pancréatique principal (50%), aspect normal du canal pancréatique principal (30%), rétrécissement du canal pancréatique principal (10%), déplacement du canal pancréatique principal (10%), Pas de dilatation distale sévère de la Wirsung, alors que l'adénocarcinome pancréatique présente toujours une dilatation modérée à sévère); L'échoendoscopie a également été largement utilisée et permet de montrer des masses pancréatiques fortement hypoéchogènes et évaluer ses rapports avec les structures avoisinantes [15].

**Le diagnostic positif repose sur l'étude anatomopathologique** d'une biopsie pancréatique percutanée guidée par l'examen échographique ou tomodensitométrique, ou d'une biopsie sous écho-endoscopie qui représente l'examen de référence [16]. L'examen anatomopathologique de la biopsie pancréatique confirme le diagnostic dans le cas présent. **Le bilan d'extension** permet l'appréciation du pronostic, et conditionne la prise en charge thérapeutique. Il doit comporter systématiquement un examen clinique minutieux, une TDM cervico-thoraco-abdominopelvienne, et une biopsie ostéomédullaire pour éliminer une forme systémique. Le reste du bilan d'extension est guidé par les signes d'appel cliniques. L'extension métastatique des LPP est caractérisée par un tropisme ostéo-médullaire, contrairement à l'adénocarcinome pancréatique dont le premier foyer métastatique est essentiellement hépatique. **Le pronostic** dépend des paramètres suivants: âge >60ans; LDH élevée; PS >1; stade IV. **La chimiothérapie** à base d'anthracycline est le traitement standard pour le LNH et comprend six à huit cycles de R-CHOP pour les patients de tous les âges. Le lymphome pancréatique de haut grade répond généralement bien à la chimiothérapie standard. Notre patient n'a pas répondu à la chimiothérapie standard RCHOP, d'où l'intérêt de faire plusieurs études pour évaluer la réponse thérapeutique aux différents protocoles de chimiothérapie. **La chirurgie** n'est pas décrite dans la littérature comme traitement de première intention des LPP. Elle ne peut être envisagée qu'en cas de difficulté diagnostique malgré la réalisation d'aspiration endoscopique guidée par ultrasons à l'aiguille fine et de biopsie percutanée, ou à visée palliative.

## Conclusion

LNH pancréatique est une tumeur rare, mais potentiellement curable, qui nécessite un diagnostic pathologique de toutes les masses pancréatiques, car son traitement et son pronostic diffèrent de ceux de l'adénocarcinome.

## Conflits d’intérêts

Les auteurs ne déclarent aucun conflit d’intérêts.
